# Rabies antibody levels in pregnant women and their newborns after rabies post-exposure prophylaxis

**Published:** 2012-03

**Authors:** Ahmad Fayaz, Susan Simani, Vida Fallahian, Ali Eslamifar, Mahboob Hazrati, Firoozeh Farahtaj, Nader Howaizi, Peivand Biglari

**Affiliations:** 1 *WHO Collaborating Center for Reference and Research on Rabies, Pasteur Institute of Iran, Tehran, Iran.*; 2 *Treatment and Prevention of Rabies Center, Department of Vaccination, Pasteur Institute of Iran, Tehran, Iran. *; 3 *Department of Clinical Research, Pasteur Institute of Iran, Tehran, Iran*

**Keywords:** *Rabies*, *Pregnancy*, *Newborn*, *Post-exposure prophylaxis*

## Abstract

**Background:** Rabies is a fatal infectious disease and rabies post-exposure prophylaxis is the method of choice for prevention of human rabies.

**Case series:** We report rabies antibody levels in cord blood and also in serum of pregnant women who were bitten by suspected animals to rabies and were immunized by purified Vero cell rabies vaccine (PVRV) and Human Rabies immunoglobulin (HRIG) serum. During the years of 2007-2010, six pregnant women by the age range of 22-35 years were admitted in treatment and prevention of rabies center in Pasture institute of Iran, in Tehran. Among them two cases were at first trimester, one at second trimester and three at third trimester of conception. The interval between biting with delivery was 5-265 days (mean 121 days).

**Conclusion:** Results of immunoglobulin illustrate that levels of rabies antibody in maternal sera with the fetus are not equal and uniform but it is proved that baby will find efficient immunity as well with minimum protective level of 0.5 IU/ml in all cases except a newborn whom had been born just 5 days after the mother’s immunization and in a shorter time than the appropriate immunization of the mother who had received her second vaccination courses.

## Introduction

Rabies is associated with 100% fatality rate among all infectious disease (1) and makes a risk for maternal death and an indeterminate risk to the fetus (2, 3). Let us review the statement of the Advisory Committee on Immunization Practices (ACIP) for rabies immunization that is applied to pregnancy and breast feeding women as well. First of all, clean the wounded site by soap and water and begin rabies post-exposure prophylaxis. Secondly, if it is an owned dog, cat or a stray animal that is available for testing, the suspected animal should be observed for 10 days. If the animal remains alive, vaccination will be ceased. Thirdly, being bitten by wild-life animals that are not available warrants initiation of post-exposure prophylaxis (PVRV and HRIG) and vaccine is administered to deltoid area (4). 

Currently, rabies post-exposure prophylaxis is a method of choice for prevention of rabies. Any pregnant woman who is bitten by stray animal is at risk. They need proper post-exposure management. Vaccination is not contraindicated in pregnancy and breast feeding. Different studies confirm that anti rabies vaccination are safe during pregnancy. One study has reported that no maternal or fetal side effects were seen among 21 pregnant women who received post exposure prophylaxis (5). 

Another study has confirmed the safety of vaccination in post-exposure pregnant women and has emphasized that treatment should never be withheld or delayed if the patient is possibly exposed to rabies (6). One case study from Mexico reported two patients with rabies exposure during the second and third trimester of pregnancy who received immunization. 

There were no side effects in mothers who were attributed to prophylaxis, which appears safe when it is given during pregnancy. Because of the fatal risk following a rabid animal bite, this case recommends that pregnancy not be a contraindication to rabies post- exposure prophylaxis (7). 

Current vaccines provide an acceptable antibody response within 7-10 days but passive immunization by immunoglobulin (with 21 days, half-life) is used for filling this 7-10 days gap (4). We have to point that rabies is endemic in Iran however safety of rabies post-exposure prophylaxis in pregnant women had been mentioned in different studies, we are going to assess the efficacy of rabies antibody in pregnant women and newborns in Iran and correlation of antibody levels between them as well. 

## Case series

We introduce six pregnant cases whom were bitten by suspected animals to rabies and referred to the Prevention and Treatment of Rabies Center, Department of Vaccination in Pasture Institute of Iran, in Tehran, Iran during 2007-10. 

Five of these cases had superficial injuries but the sixth one had superficial-to-semi deep wound. Three of these cases were bitten by dogs and the others by cats. The age range of the cases were 22-35 years, the range of gestational ages were 6-39 weeks of pregnancy. After illustrating the risk of human rabies to the patients, they accepted to receive the treatment of anti-rabies HRIG serum (in five cases), and all of them received purified Vero cell rabies vaccine (PVRV) advocated by the World Health Organization. 

Three cases that had been bitten by home-dog got three doses of vaccine on the day 0, 3, and 7 after exposure plus HRIG in two cases, but other persons who were bitten by street-cats, received 5 doses on the day 0, 3, 7, 14, and 28 plus HRIG. The interval between post-exposure immunizations and delivery was 5-265 days (Table I). 

Neither the women nor the newborns experienced any adverse effects to the serum and vaccine. The outcome of the pregnancy was satisfactory. Three cases had child birth by vaginal delivery and the other ones had cesarean section. There were no congenital anomalies in any of the infants who were born and all of them were healthy. In order to assess the efficiency of anti-rabies immunization in a mother and her newborn, we gathered blood samples from the mother and the newborn (cord blood) at the time of delivery. 

Evaluation of anti-rabies antibody levels was performed in the laboratory of Pasteur Institute of Iran. Results of anti-rabies antibodies showed that most of the cases had protective value of minimum 0.5 IU/ml except one newborn who had been born just 5 days after the mother`s immunization (by HRIG and PVRV) and in a shorter time than the appropriate period that is needed for immunization. Higher antibody levels were observed among newborns whose mothers received more doses of vaccine (Table I).

**Table I:** Characteristic parameters and levels of rabies antibody in six pregnant women

**Age (years)**	**Gestational age (weeks)**	**Interval between first vaccination and blood sampling (days)**	**Courses of vaccination up to blood sampling**	**Kind of bitten animal**	**IG level in mother blood**	**IG level in cord blood**
35	28	116	5	cat	6.3	19.8*
23	39+	5	2	cat	0.95	0.33
31	30	73	3	dog	9.8	0.5
22	6	265	5	cat	0.6	1
28	12	193	4	dog	2.4	0.8
34	25	84	5	dog	3.1	2.9

* Double checked

## Discussion

Several studies have demonstrated the safety of anti-rabies vaccination during pregnancy and there is no association between treatment and adverse outcome, and public health authorities recommend the pregnancy not be considered a contraindication to rabies post-exposure prophylaxis (2, 3). 

The policy of our department is the same: Still some clinicians and patients are reluctant to vaccination as Abazeed reported about a pregnant woman whom was exposed to bat and refused to receive treatment, but fortunately the bat was not infected (8). 

In a case study, among 21 pregnant women who received rabies post-exposure prophylaxis, no adverse effect was seen in the mother and the child (4). According to our recent report we also have not seen any serious side effects following the use of PVRV and HRIG. 

Regarding the immunogenicity of rabies post-exposure vaccination in pregnant women, Sudarshan assessed antibody levels in infants whom their mothers received PVRV during pregnancy after being bitten by suspected animals, and found that PVRV had an effective immunity in the mother and the child and it was also safe during pregnancy (9). 

Another point is that vaccination not only makes an acceptable protection, but also it causes no side effects in a mother and her child, and immunity will be achieved in the infant as well. There are many reports that confirm the safety of rabies immunization during pregnancy, but our aim was to determine the rabies antibody level and the immunization efficiency in mothers and newborns as well. 
